# Metagenomics analysis reveals universal signatures of the intestinal microbiota in colorectal cancer, regardless of regional differences

**DOI:** 10.1590/1414-431X2022e11832

**Published:** 2022-03-11

**Authors:** L. Berbert, A. Santos, D.O. Magro, D. Guadagnini, H.B. Assalin, L.H. Lourenço, C.A.R. Martinez, M.J.A. Saad, C.S.R. Coy

**Affiliations:** 1 Departamento de Medicina Interna, Universidade Estadual de Campinas, Campinas, SP, Brasil; 2 Departamento de Cirurgia, Faculdade de Ciências Médicas, Universidade Estadual de Campinas, Campinas, SP, Brasil

**Keywords:** Gut microbiota, Colorectal cancer, Faecalibacterium prausnitzii, Fusobacterium nucleatum, Dysbiosis

## Abstract

The human gut microbiota is a complex and dynamic community of microorganisms living in our intestines and has emerged as an important factor for colorectal adenocarcinoma (CRC). The purpose of our study was to investigate the microbiota composition in Brazilian CRC patients compared with a local control population (CTL) to find out which changes could be considered universal or regional features in CRC microbiota. Fecal samples were obtained from 28 CRC and 23 CTL individuals. The 16S rRNA gene was used for metagenomic analysis. In addition to the anthropometric variables, the clinical stage (TNM 2018) was considered. Patients with CRC had a significant increase in alpha diversity and a higher percentage of genus *Prevotella* and a decreased proportion of *Megamonas* and *Ruminococcus*. Additionally, the proportion of *Faecalibacterium prausnitzii* was associated with a better prognosis in the first stages of CRC, and *Fusobacterium nucleatum* proved to be an important marker of colorectal carcinogenesis and tumor aggressiveness. Although regional differences influence the composition of the microbiota, in the case of CRC, the microhabitat created by the tumor seems to be a major factor. Our results contribute to a better understanding of the carcinogenic process, and even in different environments, some factors appear to be characteristic of the microbiota of patients with CRC.

## Introduction

Colorectal cancer (CRC) is one of the most common neoplasms worldwide. The prevalence of CRC is about 4.8 million people, and the number of new cases increases each year. There are many factors involved in somatic cell transformation through the wrong path of mutations in colorectal carcinogenesis (1). The heredity component in colon cancer is between 12 and 35% (2), reflecting the environmental importance in its development. This process occurs due to the sum of genetic predisposition, disruption in immune system response, environmental damage such as through food, and microbiota alterations (3).

The human gut microbiota is a complex and dynamic community of microorganisms living in our intestines and has emerged as an important factor for CRC (4). In addition to colon cancer, disorders in the microbiota composition are associated with many diseases. Therefore, inflammatory bowel disease, type 2 diabetes mellitus, and CRC all have a common ground: they are proven to be linked with intestinal dysbiosis that results in homeostasis changes and affects local and systemic immunity, creating a chronic inflammatory environment (4). In this environment, host defenses, cell cycle, apoptosis, and anti-oxidative defenses are modulated and reactive oxygen species and nitrous oxide system production leads to DNA damage (5).

Previous data show that the geographic location of the host has the strongest association with microbiota modulation, indicating the relevance of the environment in this modulation and suggesting that CRC microbiota signature can be different in different countries and cultures (6). Recent studies indicate that although the same disease is being studied, differences in the microbiota may produce different changes that may result in a better or worse patient response (7). The Brazilian population has particularities due to its great ethnic and cultural variety and there are few studies that evaluated the microbiota in Brazilian CRC patients (8-10). In this regard, the purpose of our study was to investigate microbiota composition of Brazilian CRC patients compared with a local control population, in order to determine which changes can be considered universal or regional features of the CRC microbiota. In addition, our data can contribute to establish a possible microbiota signature that can be used as a predictor for CRC diagnosis, prognosis, and future treatment.

## Material and Methods

### Ethical statement

Subjects who agreed to participate in the study signed an informed consent form. This research was performed according to the relevant guidelines and regulations. The study was approved by the local Institutional Ethics Review Board in Brazil (CEP - Comitê de Ética em Pesquisa at Unicamp), under reference number 8.857.49/14 for the data collected from control subjects and protocol number 2.144.670/17 for the data collected from cancer patients.

### Study design and population

This was an observational cross-sectional single center study (Colorectal Unit of Campinas State University, Unicamp, Brazil) with CRC patients and control subjects (CRC, colorectal adenocarcinoma patients and CTL, subjects who underwent CRC screening with normal colonoscopy or adenomas). The following exclusion criteria were applied: current use of antibiotics or chemotherapy, adenomas with high grade dysplasia, subjects without criteria for colorectal screening colonoscopy, previous colectomy procedures, intestinal stomas, radiotherapy, inflammatory bowel disease, chronic liver disease, and familial adenomatous polyposis.

Clinical data, location, and stage of CRC (TNM 2018), history of breastfeeding and type of delivery, and morbidity were analyzed. The anatomic classification of the tumor's location was proximal colon (cecum, ascending colon, transverse colon), distal colon (descending colon, sigmoid, rectum), or synchronic. One day before colonoscopy, all participants received a liquid diet and ingested 500 mL of 10% mannitol diluted in 1000 mL of water. On the day of colonoscopy, the same nurse in the clinic collected the first stool, avoiding contamination and loss of material. All feces were solid. The feces were collected in a sterile toilet seat liner (ColOff^®^, Brazil). About 200 mg of the sample was transferred to a tube (STRATEC Biomedical AG, Germany) that preserves DNA/RNA and immediately frozen at −80°C for one week until DNA extraction.

### Metagenome profile

Total DNA of fecal samples was extracted using the Stool PSP Spin DNA kit (STRATEC Biomedical AG), an integrated system for collecting, transporting, and storing fecal samples and subsequent DNA purification. For microbiota profiling, the hyper-variable region (V3-V4) of the bacterial 16S rRNA gene was amplified following the Illumina *16S* Metagenomic Sequencing Library Preparation guide (USA), which uses the following sequence: 338F-5′TCGTCGGCAGCGTCAGTGTGTATAAGAGACAGCCTACGGGNGGCWGCAG-3 and 785R-5′ GTCTCGTGGGCTCGGAGATGTGTATAAGAGACAGGACTACHVGGGTATCTAATCC-3′ (2×300 bp paired‐end and insert size of ~550 bp).

### Bioinformatics analysis

To determinate the taxonomic composition of bacterial communities, we analyzed the V3 and V4 portion of *16S* gene rRNA using the Illumina^®^ MiSeq platform. The DNA sequencing library was built according to platform instructions. Using pared readings of 300 bp and MiSeq v3 reactors, the end of each reading was overlapped to generate high-quality full readings of the V3 and V4 region. More than 100,000 readings per sample were generated, which is sufficient for metagenomics research. The fastq sequences were analyzed using Illumina *16S* Metagenomics software (analysis software version: 2.4.60.8; reference taxonomy file: gg_13_5_species_32 bp.da), which performs taxonomic classification of the V3 / V4 region of the *16S* rRNA gene using the GreenGenes database. The analysis of gut microbiota genera was performed by the Galaxy software (open-source) and LDA-LEfSe (The Huttenhower Lab, USA), an algorithm for the identification of large biomarkers, which characterizes differences between biological conditions. The LEfSe program provides a list of the different taxa between the control group and the patient group with statistical and biological significance, classifying them according to effect size. The abundant taxa from the control group (green) or the patients (red) are given a positive or negative linear discriminant analysis (LDA) score, respectively (LDA rate >2 and significance <0.05, determined by the Wilcoxon test). LDA by effect size (LEfSe) was used to identify taxa that discriminated microbiota profiles of control and patient groups. Alpha diversity analysis was performed using the phyloseq package2 (MicrobiomeAnalyst: R version 3.6.3 (2020-02-29); web-based tool). The results were plotted across samples and reviewed as box plots for each group (11).

### Statistical analysis

The sample size was calculated based on the relative contribution of proteobacteria percentage. Assuming for α and β errors of 5% (power 95%), 26 subjects were needed in each group. The calculations were performed using G* Power software version 3.1.2 (program concept and design written by Franz University Kiel, Germany, which is freely available for Windows).

Fischer exact and the chi-squared tests were used for qualitative variables and a frequency table was built for categorical variables. Data from cancer patients and control groups are reported as means±SD or medians and interquartile range (IQR, 25-75%) for continuous variables. The Mann-Whitney U-test (non-parametric distribution) was used for comparison of continuous variables between categories. To correlate intestinal bacterial species with clinical stage disease, the Spearman test was used.

The significance level was 5% (P-value <0.05) and the SPSS v. 25.9 software (IBM Inc., USA) was used for statistical analysis.

## Results

### Study population characteristics

Between 2017 and 2018, a total of 51 subjects were included, 28 in CRC and 23 in CTL.

The baseline characteristics of the groups are detailed in Table 1. There were no significant differences between the groups regarding age (65.18±12.27, 52.04±10.08, P=0.292), body mass index (26.39±5.06, 27.14±5.52 kg/m^2^, P=0.526), and gender (P=0.75).

**Table 1:** Baseline characteristics of control (CTL) and colorectal adenocarcinoma (CRC) groups.

Variables	CRC	CTL	P-value
	n=28	n=23	
Gender (%)			
Male	50.0	65.2	0.75
Female	50.0	34.8	
Age (mean±SD)	65.18 ± 12.27	52.04 ± 10.08	0.29
BMI (kg/m^2^) (mean±SD)	26.39 ± 5.06	27.14 ± 5.52	0.52
Home location (%)			
Rural	3.6	0	1.00
Urban	96.4	100	
Delivery (%)			
Vaginal	100	87	0.08
C-section	0	13	
Last antibiotic treatment (%)			
Weeks	7.1	4.3	
1-3 months	14.3	0	
3-6 months	3.6	0	0.38
6-12 months	7.1	4.3	
More than 12 months	64.3	91.3	
Unknown	3.6	0	
Smoking Status (%)			
Current smoker	10.7	13	
Never smoker	82.1	69.6	0.62
Ex-smoker	7.1	17.4	
Breastfeeding (%)			
Until 6 months	10.7	8.7	
6-12 months	21.4	26.1	
More than 12 months	28.6	34.8	0.75
Unknown	28.6	17.4	
Never	7.1	13	
Morbidity (%)			
Diabetes	17.9	0	0.07
Hypertension	35.7	13.0	0.18
Thyroid disease	7.1	0	0.28
Dyslipidemia	7.1	4.3	1.00
Lupus	7.1	0	0.28
Diverticular disease	25.0	26.1	0.53
Tumor location (n. %)			
Proximal colon (cecum, ascending colon, transverse colon)	10 (35.7)	-	-
Distal colon (descending colon, sigmoid, rectum)	16 (57.1)	-	-
Synchronic	2 (7.2)	-	-
TNM classification (%)			
Stages 0 and I	42.9	-	-
Stage II	25.0	-	-
Stage III	28.6	-	-
Stage IV	3.6	-	-

Data were compared by Fischer exact test, chi-squared test, or Mann-Whitney U-test. TNM: tumor, node, metastasis.

Distal colon (57.1%) was the most frequent CRC location. Regarding TNM classification, early stages were the most common, accounting for 42.9% of the sample (stages 0 and I), stage II 25%, stage III 28.6%, and stage IV 3.6%.

### Intestinal microbiota analysis

**Figure 1:**
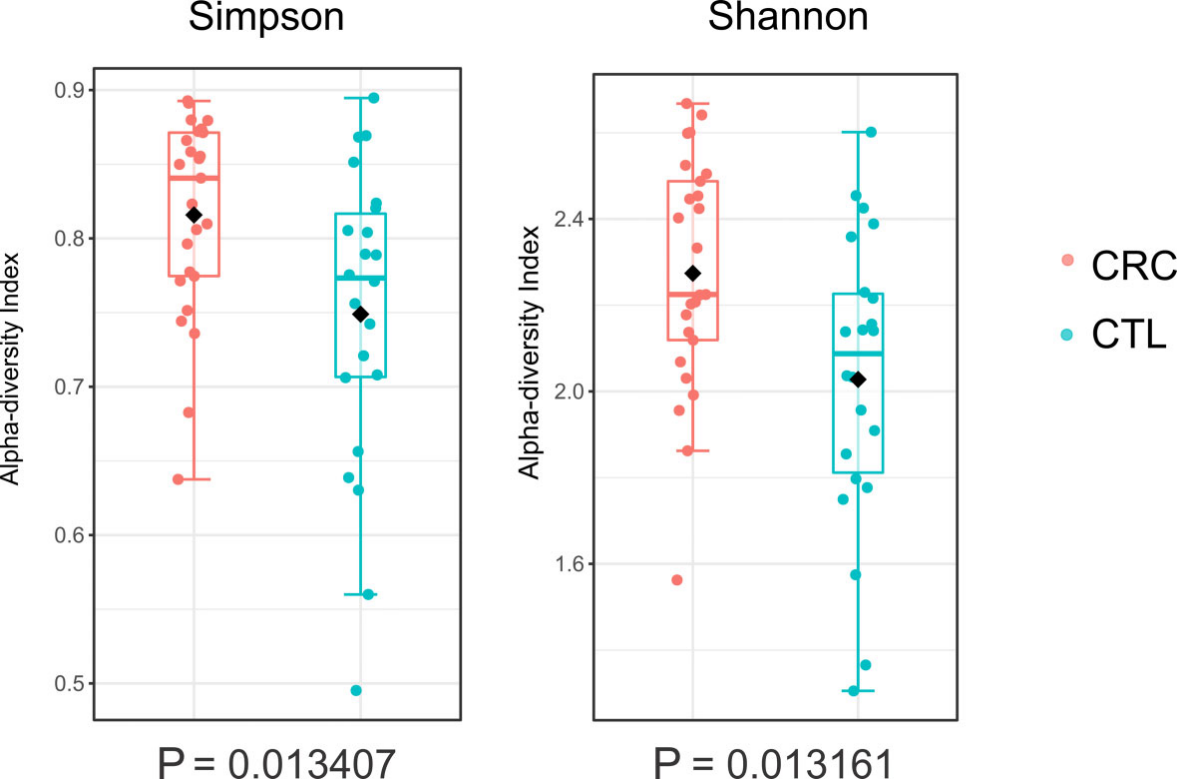
Comparison of alpha diversity of the colorectal adenocarcinoma (CRC) and the control (CTL) groups using the Shannon and Simpson indexes. Boxplots showing the median and interquartile range of each group, and each point corresponds to an individual's alpha diversity. Mann-Whitney U-test.

**Figure 2:**
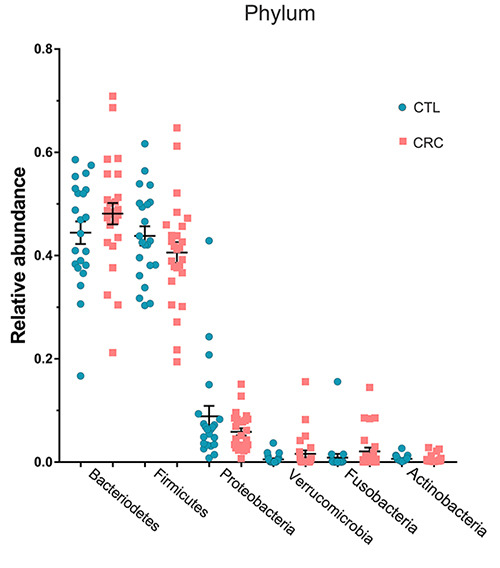
Relative abundance of bacterial phyla. Comparison of metagenomics analysis of bacterial phyla from gut microbiota in colorectal adenocarcinoma (CRC) patients (n=28) and control (CTL) subjects (n=23). The data were obtained from sequencing of the hyper-variable region (V3-V4) of the bacterial *16S* rRNA gene. Points represent the relative abundance of each participant.

Regarding alpha diversity (Figure 1), analyzed with Simpson and Shannon models, CTL had greater diversity (P<0.01). Both models consider the number of present species and the relative abundance of each species (Simpson's values vary between 0 and 1 and Shannon's between 1.5 and 3.0). There was no significant difference in the proportion of the main bacterial phyla (Figure 2).

**Figure 3:**
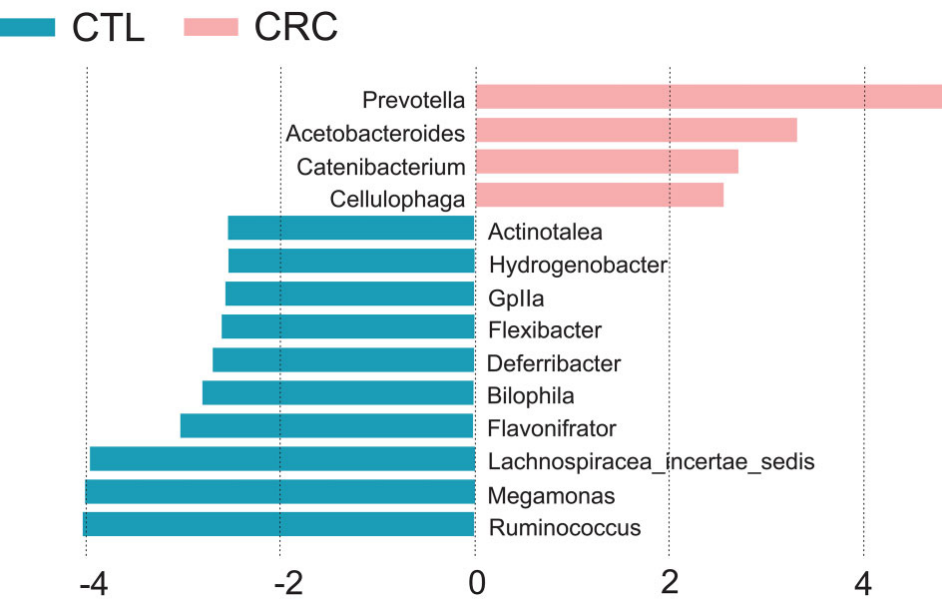
Linear discriminants analysis (LDA) associated with effect size (LEfSe) showing differences in genus between the colorectal adenocarcinoma (CRC) patients and control (CTL) subjects (rate with an LDA score >2.5 and a significance of <0.05 determined by the Wilcoxon signed rank-test).

LEfSe (Figure 3) results indicated genus differences between groups (rate with an LDA score >2 and a significance of <0.05, Wilcoxon signed rank-test) with *Prevotella* predominance in CRC and *Megamonas* and *Ruminococcus* predominance in CTL.

There were no differences between groups regarding the species *Akkermansia muciniphila*, *Faecalibacterium prausnitzii*, *Lachnospira pectinoschiza*, *Peptostreptococus anaerobius*, *Escherichia coli*, *and Enterococus faecalis.*

**Figure 4:**
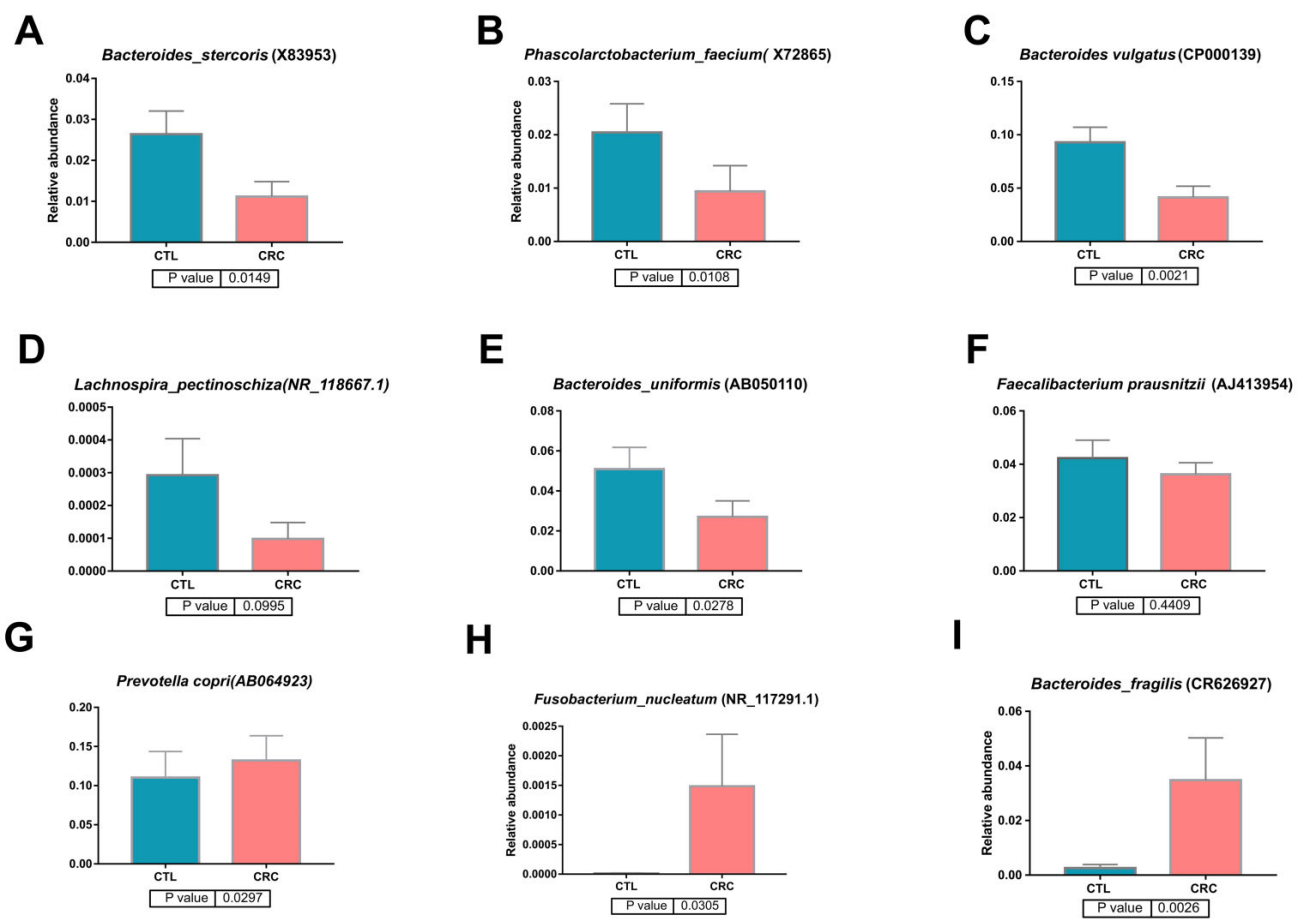
Comparison of the relative abundance of bacterial species in the intestinal microbiota of the control (CTL) and colorectal adenocarcinoma (CRC) groups.

Higher amounts of *Prevotella copri* (P=0.029), *Bacteroides fragilis* (P=0.032), and *Fusobacterium nucleatum* (P=0.03) were observed in CTL and a greater abundance of *Bacteroides vulgatus* (P=0.002), *Bacteroides stercoris* (P=0.01), *Bacteroides uniformis* (P=0.02), and *Phascolarctobacterium faecium* (P=0.01) occurred in CRC (Figure 4).

**Figure 5:**
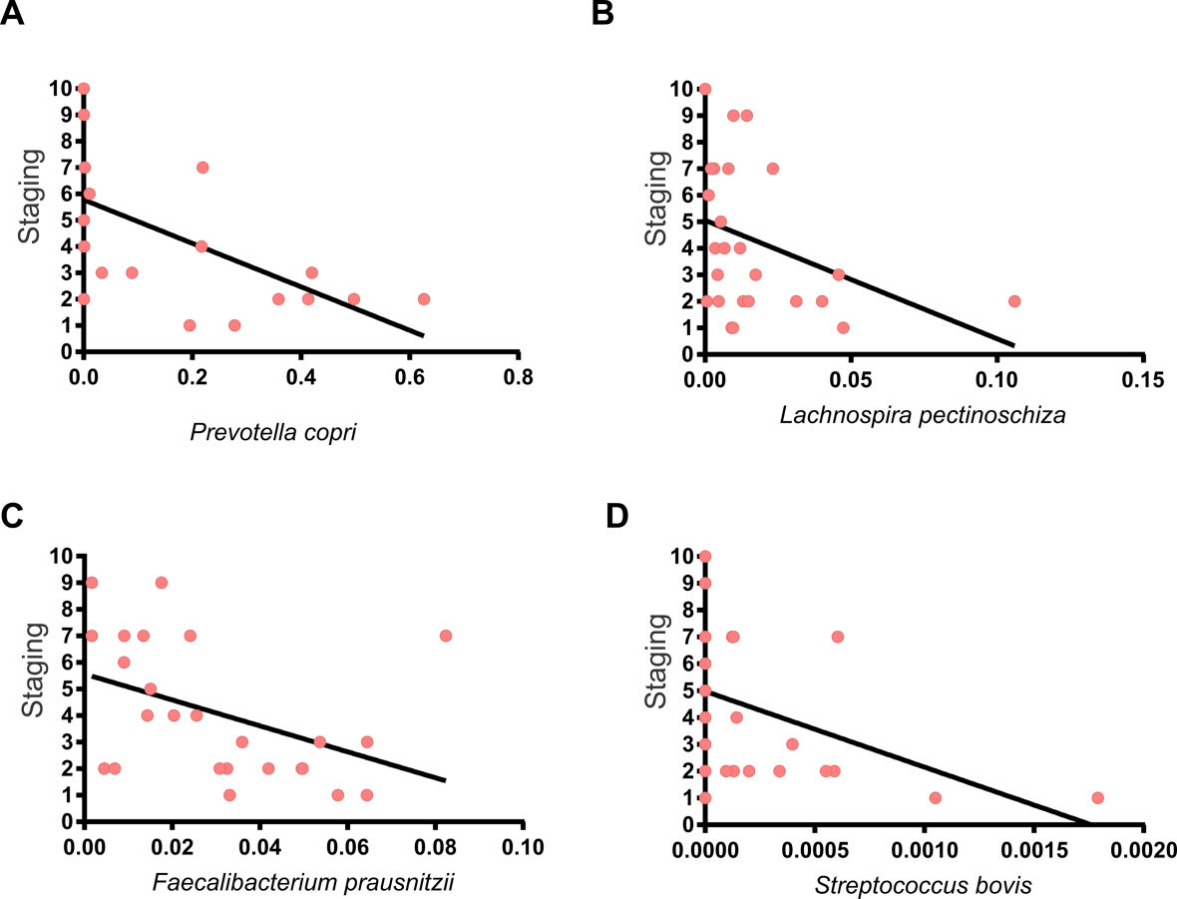
Spearman's correlation between cancer staging and bacterial composition. Inverse correlation between cancer staging and **A**, *Prevotella copri* (Spearman R=-0.5866 P=0.003); **B**, *Lachnospira pectinoschiza* (Spearman R=-0.4222 P=0.041); **C**, *Faecalibacterium prausnitzii* (Spearman R=-0.488 P=0.016); and **D**, *Streptococcus bovis* (Spearman R=-0.482 P=0.012).

There was an inverse correlation between cancer stage and *Prevotella copri* (Spearman R=-0.5866, P=0.003), *Lachnospira pectinoschiza* (Spearman R=-0.4222 P=0.041), *Faecalibacterium prausnitzii* (Spearman R=-0.488 P=0.016), and *Streptococcus bovis* (Spearman R=-0.482 P=0.012), i.e., the higher the clinical stage, the lower the amount of these species (Figure 5).

## Discussion

Our study found that Brazilians with CRC have an altered gut microbiota composition, characterized by increased alpha diversity and different amounts of some genera and species.

Similar to our results, other Brazilian studies have also found an increase in alpha diversity in CRC biopsy samples (8,10). In the same way, *B. fragilis*, a symbiotic organism common in the human intestinal tract, was found to be more abundant in tumor samples (12) and CRC stool samples (8). This species can adhere to the inflamed mucosal surface of patients with colon cancer, alter intestinal permeability, and increase metastatic potential (13). There are two subtypes, one of which is enteropathogenic (14). The toxin released by this subtype (*Bacteroides fragilis* toxin) increases cell proliferation, the release of pro-inflammatory factors by the colonic epithelium, and damage to DNA (15).

Although the population studied by de Carvalho et al. (10), Thomas et al. (8), and our study was from the Brazilian state of São Paulo, the abundance of some genera was not similar in the three studies. Thomas et al. (8) and de Carvalho et al. (10) showed that the genus *Odoribacter* was increased in the CRC group (8,10), which was not found in our samples. The genus *Ruminococcus* was found depleted in the CRC group by de Carvalho et al. and our study but not by Thomas et al. Similarly, de Carvalho et al. (10) and Thomas et al. (8) diverged in the abundance of other genera. These differences may be due to the*16S* rRNA region of choice on the bacterial community for sequencing the *16S* gene (16).

Similar results regarding alpha diversity were shown among groups in a large meta-analysis from 5 countries that included 413 subjects with CRC, 143 adenomas, and 413 controls (17). In contrast, some studies have described a decrease in alpha diversity associated with CRC in stool samples (18). In an Austrian study, the alpha diversity showed no difference between healthy control subjects with advanced adenomas and CRC patients (19).

Individuals with CRC have a higher percentage of genera *Prevotella* and *Acidaminobacter* and a relatively decreased proportion of *Megamonas* and *Ruminococcus*. *Prevotella* has already been associated with increased production of IL-17 in the mucosal cells of patients with CRC (20,21). *Acidaminobacter* was also found to be over-represented in CRC stool samples (22). *Ruminococcus* genera are related to the fermentation of complex carbohydrates and producers of short-chain fatty acids. This genus and *Megamonas* were increased in the control group in our study, which is in line with other studies (19).

In a cohort study with healthy controls and CRC patients from the United States and Canada, there was an increase in *Fusobacterium* and *Porphyromonas* and a decrease in *Bacteroides* (23). The CRC group had increased *Prevotella copri*, *Bacteroides fragilis,* and *Fusobacterium nucleatum* species. In the control group, there was a predominance of *Bacteroides vulgatus*, *Bacteroides stercoris,* and *Bacteroides faecium* species. The increase of *Prevotella copri*, *Lachnospira pectinoschiza*, *Faecalibacterium prausnitzii*, and *Streptococus bovis* was associated with early cancer stages

*Streptococcus bovis* (*Streptococcus gallolyticus*) was the first species described in the literature to be related to CRC. McCoy and Mason (24) reported endocarditis due to this species in a patient with a colon tumor in 1951. The current study showed a correlation of *S. bovis* with early disease stages. This finding may be related to the CRC individuals having a higher proportion of initial tumors and all cases being located in the proximal colon. Therefore, it should not be assumed that the presence of *S. bovis* is associated with less tumor aggressiveness, but only with its proximal location. Results similar to those reported in the literature are also expected for tissue samples, as a higher prevalence was observed when the microbiota was analyzed from biopsies rather than fecal samples (25). The hypothesis is that such a species can adhere to the tissue and induce a pro-inflammatory environment that can lead to tumor progression, especially in pre-neoplastic lesions (26).

In our study, larger amounts of *Prevotella copri* and *F. prausnitzii* were also observed in the early stages of disease. *Prevotella copri* has a controversial role in human health (27). Some articles relate this species to vegetarian and fiber-rich diets, suggesting that *P. copri* helps in fiber degradation and related to health condition (14,28). It is associated with the production of short-chain fatty acids, which is the substrate that nourishes enterocytes and has anti-inflammatory effects (29). In contrast, other authors have shown that the increase in the amount of *P. copri* is related to inflammatory conditions, such as rheumatoid arthritis (30) and insulin resistance (31). These controversial results can be justified by the variation in genotypes of this species, which is mainly modulated by diet, as demonstrated by De Filippis et al. (21). Furthermore, bacteria of this genus have been associated with CRC (32).

In contrast, *Faecalibacterium prauznitzii* is a butyrate-producing bacteria, being considered the most important of the human intestinal microbiota, commonly associated with health status (29,33). Clinical staging is currently the most important indicator of prognosis in patients with CRC. However, new strategies to identify prognostic predictors are being investigated. The species *F. prausnitzii* was found in greater quantity among patients who had longer postoperative survival (34) and can be a marker of lower aggressiveness.

Finally, *Fusobacterium nucleatum* was shown to be an important marker of colorectal carcinogenesis and tumor aggressiveness (35). It is known that *F. nucleatum* can adhere to the epithelium, and when it invades, it recruits immune cells and creates an inflammatory environment by modulating the response of T cells and promoting metastasis (36,37). A Brazilian study found more *F. nucleatum*and C*lostridium difficile* in the *CRC* fecal samples (9). de Carvalho et al. showed higher quantities of *F. nucleatum* in tumor tissue, which was associated with more undifferentiated invasive proximal tumors, loss of expression of MLH1 and MSH2 PMS2, and worse prognosis (10). In a study in Sweden using combined tests for *Escherichia coli* and*F. nucleatum*, CRC was detected with a specificity of 63.1% and a sensitivity of 84.6% (38). Similarly, our findings were compatible with current data and confirmed that *F. nucleatum* can be considered an important marker of colorectal carcinogenesis and tumor aggressiveness, since alterations in tumor environment may favor proliferation of opportunistic bacteria (39). As seen previously, this species is found in greater numbers in patients with CRC around the world and in Brazil. Zeller et al. (40), using a similar method, evaluated French subjects and found that *F. nucleatum* was one of the four most important species correlated with cancer diagnosis.

Our study had limitations. First, the sample size was small, and the cross-sectional design did not allow determination of cause-effect relationships. In addition, to understand the functional features of the species, all its genetic compounds must be analyzed, which is possible using the shotgun method rather than by 16S RNA. Another characteristic of this sample that may have affected the results was that almost 43% were early-stage tumors.

### Conclusions

We have demonstrated that gut dysbiosis is associated with CRC. Patients with CRC had a significant increase in alpha diversity and a higher percentage of the genus *Prevotella* and a decreased proportion of *Megamonas* and *Ruminococcus*. Additionally, the proportion of *F. prausnitzii* was associated with a better prognosis in the first stages of CRC, and *Fusobacterium nucleatum* proved to be an important marker of colorectal carcinogenesis and tumor aggressiveness. Although regional differences influence the composition of the microbiota, the microhabitat created by CRC seems to be a major factor. Our results contribute to a better understanding of the carcinogenic process, and, even in different environments, some factors appear to be characteristic of the microbiota of patients with CRC.
